# The influence of process and patient factors on the recall of consent information in mentally competent patients undergoing surgery for neck of femur fractures

**DOI:** 10.1308/10.1308/003588412X13171221591970

**Published:** 2012-07

**Authors:** SK Khan, K Karuppaiah, AS Bajwa

**Affiliations:** South Tees Hospitals NHS Foundation Trust,UK

**Keywords:** Informed consent, Neck of femur fractures, Abbreviated mental test score

## Abstract

**INTRODUCTION:**

Informed consent is an ethical and legal prerequisite for major surgical procedures. Recent literature has identified ‘poor consent’ as a major cause of litigation in trauma cases. We aimed to investigate the patient and process factors that influence consent information recall in mentally competent patients (abbreviated mental test score [AMTS] ≥6) presenting with neck of femur (NOF) fractures.

**METHODS:**

A prospective study was conducted at a tertiary unit. Fifty NOF patients (cases) and fifty total hip replacement (THR) patients (controls) were assessed for process factors (adequacy and validity of consent) as well as patient factors (comprehension and retention) using consent forms and structured interview proformas.

**RESULTS:**

The two groups were matched for ASA (American Society of Anesthesiologists)****grade and AMTS. The consent forms were adequate in both groups but scored poorly for validity in the NOF group. Only 26% of NOF patients remembered correctly what surgery they had while only 48% recalled the risks and benefits of the procedure. These results were significantly poorer than in THR patients (*p*=0.0001).

**CONCLUSIONS:**

This study confirms that NOF patients are poor at remembering the information conveyed to them at the time of consent when compared with THR patients despite being intellectually and physiologically matched. We suggest using pre-printed consent forms (process factors), information sheets and visual aids (patient factors) to improve retention and recall.

The ‘informed consent’ philosophy treats the patient as an autonomous individual who is presented with complete, evidence-based information about the risks and benefits of an intervention and makes a rational choice without being subjected to duress.^1,2^ Consent is a two-way process. It is not a one-off event but should be thought of as a ‘continuum’ unfolding across a patient’s total duration of care.^1^ The healthcare professional proposing and performing the procedure is ultimately responsible for taking the patient’s consent. However, this can be delegated to a person who is appropriately trained, suitably qualified and has specific knowledge of the procedure including its risks.^2^ Consent for any surgical procedure is mandated by the General Medical Council and the surgical royal colleges.^2,3^

Fractures involving the neck of femur (NOF) comprise a high volume emergency presentation to trauma departments with a national incidence of 76,000 per year^4^ although this is expected to rise to 100,000 by 2033.^5^ It is a high risk entity with a six-month mortality rate of 6–10% and a one-year mortality rate of 22–29%.^6–8^ It is also a high cost practice, with the current NHS expenditure estimated at £1.4 billion in treatment costs alone.^4^

Surgical treatment of hip fractures is not free of intra-operative or post-operative risks either. Data from the NHS Litigation Authority (NHSLA) show 12 cases of intra-operative negligence or poor surgical outcome in hip fracture surgery from 2000 to 2006. The average payment for these interventions was equivalent to $120,820, nearly 0.5% of the total payouts during this period.^9^ ‘Poor consent’ was the second most common generic cause for litigation in this period for all types of trauma and elective cases. For these clinical, ethical, economic and medicolegal reasons, it is imperative that the consent process is standardised and that it is truly informed.

In most NHS hospitals, the consent in all trauma cases is taken predominantly by the most junior members of the team.^10,11^ Previous studies have reported the inadequacy of the consent process in NOF patients.^12–14^ The adequacy and validity of consent are, however, not surrogates for the patient’s comprehension and retention of the presented information, which are purely ‘patient’ factors. It is essential to differentiate between ‘process’ and ‘patient’ factors in informed consent as these can both affect the recall of this information. While the process factors have been described in detail,^10,12–14^ we have not come across any studies looking at patient factors in hip fracture patients.

The primary aim of this study was to assess these patient factors (comprehension and retention) and the resultant recall of consent information in a mentally competent group of hip fracture patients. These were compared with an intellectually matched group of patients undergoing primary total hip replacement (THR) for osteoarthritis. A secondary aim was to assess the process factors (adequacy and validity of consent).

## Methods

A prospective case-control study was conducted over two months in the trauma and elective orthopaedic units of a university teaching hospital after obtaining ethics approval. The cases comprised eligible patients with hip fractures (NOF group) while a matched group of patients undergoing THRs were recruited as controls (THR group). The controls were matched for physiology and co-morbidities as stratified by the ASA (American Society of Anesthesiologists) grade and also for intellectual capacity as determined by their abbreviated mental test score (AMTS). Sample size calculations were performed and the power of the study was set at 80%. The significance level was set at 5% (*p*<0.05). Recall of the consent process was used as the principal outcome measure. It was estimated that 50 patients in each arm were required.

### Neck of femur fracture group

All patients with NOF fractures admitted consecutively to the trauma unit were assessed for inclusion until 50 eligible and consenting patients were recruited. These were defined as proximal femoral fractures, comprising both extracapsular and intracapsular fractures (AO/ASIF [Arbeitsgemeinschaft für Osteosynthesefragen/Association for the Study of Internal Fixation] types 31-A and 31-B). Exclusion criteria included patients with an AMTS of <6, those with periprosthetic fractures and those admitted for revision surgery.

### Total hip replacement group

All patients with hip osteoarthritis being admitted consecutively to the elective unit for a primary THR were considered for inclusion until 50 eligible and consenting patients were recruited. Exclusion criteria included patients with an AMTS of <6, those having arthroplasty for post-traumatic complications and those admitted for revision arthroplasty.

## Methods

Two different tools were used in all patients in both groups. First, the consent forms were assessed for process factors including adequacy (timing of the consent, grade of consenting doctor, completeness and veracity of information) and validity (legibility, patient’s signature and consultant verification). The last page of the consent form consisted of the operation note, which was checked for date of surgery, type of procedure and grade of the operating surgeon.

A formalised questionnaire proforma was then used for semi-structured interviews on the first post-operative day. The proforma included questions related to patient factors and also allowed for recording of qualitative responses. (A copy of the proforma is available from the corresponding author on request.) Each interview began with an evaluation of the AMTS and the ASA grade was noted from the anaesthetic record for that procedure. Patients were asked if they remembered what procedure was performed, what they understood about the procedure and its associated risks, and whether they remembered being told of these risks. Care was taken to conduct the interviews 24 hours post-operatively to reduce the effects from a general anaesthetic and at least 3 hours since the last dose of opioid analgesia.

The data were analysed using InStat® (GraphPad Software, La Jolla, CA, US). Differences between the two groups were analysed using suitable t-tests for parametric variables and Fisher’s exact test for binomial variables.

## Results

### Process factors

In the NOF group (*n*=50), 27 patients (54%) had sustained extracapsular fractures and had their fractures stabilised with either dynamic hip screws (*n*=20, 74%) or proximal femoral nails (*n*=7, 16%). Twenty-three patients (46%) sustained intracapsular fractures and were treated with a hemiarthroplasty (*n*=18, 78%) or THR (*n*=5, 22%). Forty-nine consent forms (98%) were filled in by core trainees (CTs) and one (2%) by a specialty trainee (ST). Seven patients (14%) received a copy of their consent forms. The risks and benefits of the relevant operations were documented on 42 consent forms (84%). Consent forms were signed and dated by both the consenting clinician and the patient in 42 cases (84%). Forty-one procedures (82%) were carried out by CTs or STs and nine operations (18%) were performed by consultants.

The THR group included 26 right-sided and 24 left-sided procedures. Consent was obtained by consultants in 34 (68%), STs in 13 (26%) and CTs in 3 (6%) cases. Sixteen patients (32%) received their copies of the consent forms. The risks and benefits of the procedure were documented in detail on 42 consent forms (84%). All 50 consent forms were signed and dated by both the consenting clinician and the patient (Table 1).

**Table 1:** Process factors in the neck of femur (NOF) fracture and total hip replacement (THR) groups

	NOF	THR	Statistical tests
Risks and benefits documented, veracity of information, timing (adequacy)	Yes: 84% (*n*=42) No: 16% (*n*=8)	Yes: 84% (*n*=42) No: 16% (*n*=8)	*p*=1.0*
Legibility, signed and dated by both clinician and patient (validity)	Yes: 84% (*n*=42) No: 16% (*n*=8)	Yes: 96% (*n*=48) No: 4% (*n*=2)	*p*=0.008*
Patient copy	Yes: 14% (*n*=7) No: 86% (*n*=43)	Yes: 32% (*n*=16) No: 68% (*n*=34)	*p*=0.004*

* Fisher’s exact test

### Patient factors

There were 39 (78%) women and 11 (22%) men in the NOF group. Their age range was 54–93 years (mean: 79.3 years, standard deviation [SD]: 10.4 years). The AMTS ranged from 6 to 10 (mean: 7.9, SD: 1.4) and the ASA grade ranged from 1 to 4 (mean: 2.6, SD: 0.7). Only 13 patients (26%) remembered correctly what surgery they had undergone while 37 (74%) described the wrong procedure. Twenty-four patients (48%) could remember the pertinent risks and possible complications discussed with them at the time of the consent while 26 (52%) had no recollection of any risks being discussed with them. Forty-eight patients (96%) said they would still have the surgery if they were informed of all the risks involved.

The THR group included 26 (52%) women and 24 (48%) men. Their age range was 46–81 years (mean: 66.6 years, SD: 8.9 years). The AMTS ranged from 6 to 10 (mean: 8.0, SD: 1.2) and the ASA grade ranged from 1 to 3 (mean: 2.3, SD: 0.5). All 50 THR patients were able to recall the nature of their procedure with the possible risks and all of them indicated they were willing to have it performed again (Table 2).

**Table 2:** Patient factors in the neck of femur (NOF) fracture and total hip replacement (THR) groups

	NOF	THR	Statistical tests
*Demographics*			
Sex	Male: 22% (*n*=11)	Male: 48% (*n*=24)	*p*=0.01*
	Female: 78% (*n*=39)	Female: 52% (*n*=26)	
Age (years)	Mean: 79.3	Mean: 66.6	*p*<0.0001**
	SD: 10.4	SD: 8.9	95% CI: 8.858–16.542
	Range: 54–93	Range: 46–82	
AMTS	Mean: 7.9	Mean: 8.0	*p*=0.7**
	SD: 1.4	SD: 1.2	95% CI: -0.617–0.417
	Range: 6–10	Range: 7–10	
ASA grade	Mean: 2.6	Mean: 2.4	*p*=0.1**
	SD: 0.7	SD: 0.5	95% CI: -0.041–0.441
	Range: 1–4	Range: 1–3	
*Recall by patients*			
What surgery?	Correct: 26% (*n*=13)	Correct: 100% (*n*=50)	*p*=0.0001*
	None or incorrect: 74% (*n*=37)	None or incorrect: 0% (*n*=0)	
Knowledge of complications	Yes: 48% (*n*=24)	Yes: 100% (*n*=50)	*p*=0.0001*
	No: 52% (*n*=26)	No: 0% (*n*=0)	
Would knowledge of these complications change their decision?	Yes: 4% (*n*=2)	Yes: 4% (*n*=2)	*p*=1.0*
	No: 96% (*n*=48)	No: 96% (*n*=48)	

CI = confidence interval; AMTS = abbreviated mental test score; ASA = American Society of Anesthesiologists

* Fisher’s exact test

** t-test

## Discussion

The information provided to hip fracture patients at the time of consent is variable and not always standardised or reasonably adequate.^12–14^ The most frequent and consistently communicated possible risks include infection, venous thromboembolism, neurovascular damage, anaesthetic risks, chest infection, myocardial infarction and scar problems. Mechanical complications such as dislocation, malunion, non-union, avascular necrosis, leg length discrepancy and periprosthetic fractures tend to be less frequently mentioned.

The Department of Health consent guide recommends that the seeking and giving of consent is usually a process rather than a one-off event.^1^ For major procedures, it is good practice to obtain consent some time before the procedure. This would give the patient enough time to weigh up the information presented and to make an informed decision.^15^ This is, however, not always feasible in NOF fractures due to the sudden nature of the injury and the need to operate as quickly as possible so as to reduce morbidity and mortality.^16–18^ THR patients, on the other hand, have much more time to process information, check it with other sources (eg friends, general practitioners or the internet), receive a second consultation and make a final decision.

The consent forms were adequate in both groups but scored poorly for validity in the NOF patients. One method to improve these is to convey standardised information using procedure specific consent forms.^19^ This approach has been reported to improve recall rates in patients undergoing knee arthroscopy and arthroplasty.^20^ The variability of the information recorded on ordinary consent forms by junior staff lends support to the use of such printed and standardised consent forms.^12,20,21^ This would also resolve typographical issues such as legibility, corrections, overwriting and the use of abbreviations.

The present study identified that only 26% of NOF patients remembered correctly what surgery they had undergone while only 48% of them could recall the pertinent risks and possible complications being discussed with them at the time of the consent. This is significantly worse than the consent retention in the THR group.

Patients generally tend not to question much when an intervention is proposed to them. Mahadevan and Gupta reported that 75% of orthopaedic patients signed consent forms willingly despite not fully understanding details of the procedure and the risks (eg they did not understand what a deep vein thrombosis was).^22^ This becomes more relevant in hip fractures as the patients are elderly, have less basic knowledge and are distressed by pain on admission. Moreover, they can be confused from the administered analgesia and may be apprehensive or anxious in an unfamiliar environment. Purohit and Kalairajah^21^ investigated 64 hip fracture patients with an AMTS of >1 and scored their comprehension of the consent using an adapted questionnaire.^23^ The average first post-operative day ‘comprehension score’ was 8.7 out of a possible 14 and a strong correlation was observed between the AMTS and consent comprehension. Bhangu *et al* assessed recall in 20 hip fracture patients and found that 85% could recall the name of the operation but only 26% remembered the risk of complications.^24^

It has been shown that patients remember as little as 20% of the information given to them during a five-minute consultation.^25^ Information retention can be increased to 50% if there is additional written information.^26–29^ The use of information sheets has been shown to improve recall in orthopaedic patients undergoing elective procedures. Patients receiving information sheets at pre-assessment also scored significantly higher on questioning at the time of their arthroplasty procedures in Langdon *et al’*s randomised trial.^30^ Similarly, Ashraff *et al* reported a significantly better recall of consent information in elective orthopaedic patients who were given leaflets than those who were not.^31^ The 100% recall rate in the THR group in our study probably owes to the visual aids and written sheets used in group-based, pre-operative ‘arthroplasty classes’ held at our unit.

We acknowledge the limitations of this study. The two groups were not matched for age. This is unavoidable, given that hip fractures are generally fragility fractures in an older age group while elective arthroplasty patients seek surgery at an earlier age to regain their mobility and quality of life. We defined mental competence as an AMTS of 6 or more although the available literature supports a cut-off value of 7. We did not construct a model for assessing the patient’s comprehension of the consent information and could therefore not score it objectively. The grades of the consenting clinicians could not be controlled as a higher proportion of patients coming to an elective hip replacement clinic would be seen and consented by consultants compared with hip fracture patients. Lastly, no adjustment was made for patients who had their surgery under general anaesthesia versus those who had spinal anaesthesia.

## Conclusions

**Figure 1:**
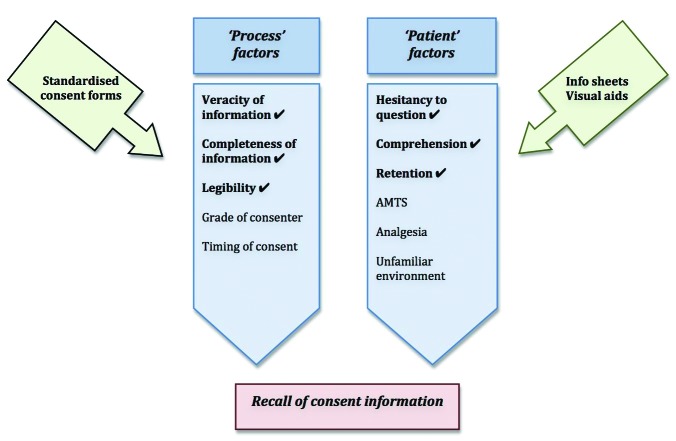
The process and patient factors in consent and how these can be influenced

**Figure 2:**
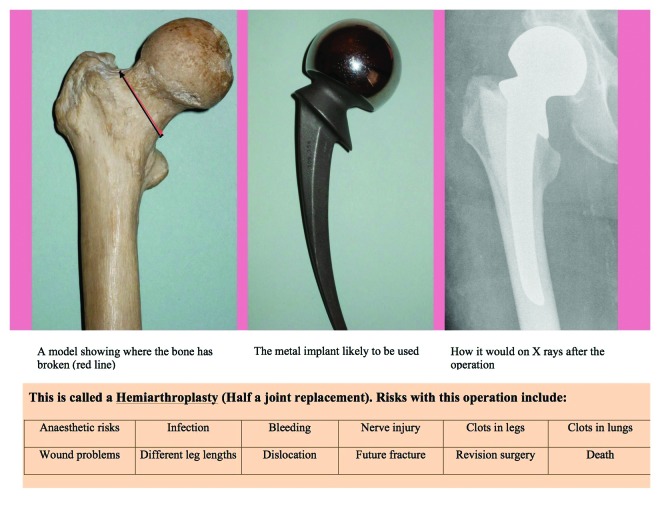
Example of a memory aid for a patient being treated with a hemiarthroplasty

This study confirms that hip fracture patients are poor at recalling the information conveyed to them at the time of consent for their respective procedures compared with THR patients. We propose using procedure specific pre-printed consent forms, information sheets and visual aids as part of a multipronged strategy when seeking consent from these patients (Fig 1). The standardised consent forms would convey accurate and precise information to educate patients and enable comprehension while the information sheets and visual aids would reinforce this information to improve recall. We suggest these sheets or aids be laminated, with a simple and visual summary of the procedure and its possible risks. An example of a memory aid for a hip hemiarthroplasty is shown in Figure 2.

AMTS = abbreviated mental test score

We did not come across any mentally competent patient in our study who did not wish to be informed of the risks involved in hip fracture surgery. We do not therefore support the notion that too much information can be distressing for this group of patients.
